# Characteristics and clinical challenges in patients with substance use disorder in palliative care—experience from a tertiary center in a high-income country

**DOI:** 10.1186/s12904-024-01366-x

**Published:** 2024-01-30

**Authors:** Laura Marti, Ellen Hünerwadel, Bigna Hut, Sebastian M. Christ, Fabienne Däster, Markus Schettle, Annina Seiler, David Blum, Caroline Hertler

**Affiliations:** 1 University of Zurich, Zurich, Switzerland; 2 Competence Center Palliative Care, Department of Radiation Oncology, University Hospital Zurich, Rämistrasse 100, Zurich, 8091 Switzerland; 3 Department of Radiation Oncology, University Hospital Zurich, Zurich, Switzerland

**Keywords:** Palliative care, Vulnerable groups, Substance use disorders, Challenges, Symptoms

## Abstract

**Background:**

Access to palliative care is often limited for challenging and vulnerable groups, including persons with substance use disorders. However, with optimized healthcare options and liberal substitution policies, this patient group is likely to increase over the upcoming years, and comorbidities will also influence the need for palliative support. Here, we aim at analyzing characteristics and specific challenges associated with substance use disorders (SUD) in palliative care.

**Methods:**

We retrospectively reviewed all patients diagnosed with substance use disorder that were treated at our Competence Center Palliative Care within the University Hospital Zurich, Switzerland between 2015 and 2021. Patient characteristics, including age, gender, duration of hospitalization, as well as specific metrics like body mass index, distinct palliative care assessment scores, and in-hospital opioid consumption were retrieved from the electronic patient files. Demographics and clinical data were analyzed by descriptive statistics, and compared to those of a control group of palliative care patients without SUD. An opioid calculator was used to standardize opioid intake based on morphine equivalents for meaningful comparisons.

**Results:**

The primary characteristics revealed that the majority of individuals were single (56%), had no children (83%), lived alone (39%), and were either unemployed or recipients of a disability pension (in total 50%). Nicotine (89%), opioids (67%), and alcohol (67%) were the most used substances. We identified various comorbidities including psychiatric illnesses alongside SUD (56%), hepatitis A, B, or C (33%), and HIV infection (17%). Patients with SUD were significantly younger (*p* < 0.5), predominantly male (*p* < 0.05), and reported a higher prevalence of pain (*p* < 0.5) compared to the standard cohort of palliative patients. Regarding the challenges most frequently reported by healthcare practitioners, non-compliance, multimorbidity, challenging communication, biographical trauma, lack of social support, and unstable housing situations played a key role.

**Conclusion:**

Patients with SUD represent a complex and vulnerable group dealing with multiple comorbidities that profoundly affect both their physical and psychological well-being. Understanding their unique characteristics is pivotal in providing precise and suitable palliative care.

**Supplementary Information:**

The online version contains supplementary material available at 10.1186/s12904-024-01366-x.

## Background

Substance use disorders (SUD) are a leading cause of worldwide disease burden, and are associated with high morbidity and mortality (Whiteford et al.). While the prevalence of opioid dependence is reportedly high in Western Europe [1], alcohol still remains the most harmful substance followed by heroin and cocaine [2]. Accordingly, approximately 250 000 persons in Switzerland are reportedly alcohol dependent. Moreover, 40 000 persons are reported to be in substance abuse counselling for any International Statistical Classification of Diseases (ICD)-11 F-coded SUD.

Several high-income European countries, including Switzerland, allow for access to interventions reducing the risk of mortality especially from opioid dependence, including substitution treatment and treatment of associated transmitted diseases such as Human Immunodeficiency Virus (HIV). Still, while these interventions reduce the overall mortality, patients with SUD are still prone to suffer from comorbidities and secondary diseases that eventually lead to a high symptom burden and a need for palliative support.

As a result, the great majority of palliative care clinicians will encounter patients with SUD. Thus, it is important to understand and characterize this group of patients, with the goal to allow for an optimal patient care, and good information for the care team [3]. In this context, it is important to acknowledge the role of dual diagnoses, the co-appearance of SUD with another psychiatric disease. Indeed, in Switzerland, approximately 1/5 of psychiatric hospitalisations were associated with SUD. This duality adds to the care burden, and the care system often lacks knowledge, sufficient training, and apprehension to care for people with addiction and the subsequent consequences [3]. To date, there are no uniform guidelines for the treatment of SUD in people with life-threatening diseases [4]. Addressing addiction directly remains a taboo in clinical daily life because practitioners may be fearful to appear judgmental [5]. Therefore, the need for primary SUD treatment skills for PC clinicians might be similar to the requirements for specialists such as oncologists to learn primary PC skills. The fact that communication with SUD patients remains demanding for palliative care teams rather reflects lack of specific training and insecurity than social hostility. Therefore, more information on how to treat and understand these patients is warranted.

Here, we report on the experiences with SUD patients treated at our Competence Center Palliative Care. We characterize these patients and their needs as well as the disease-specific challenges for the team, with the overall aim to identify areas of improvement for comprehensive treatment.

## Material & methods

### Data source

We conducted a retrospective single center cohort study of patients > 18 years suffering from SUD according to ICD-11 that presented at the Competence Center Palliative Care at the University Hospital Zurich (USZ) between 2015 and 2021 and identified 18 patients. A group of 377 palliative care patients without a history of SUD was used as control cohort. Electronic patient files were the primary source of data acquisition.

### Patient characteristics

We obtained data on patient demographics, primary disease, symptom load and distress levels. In addition, we collected data on advance directives, network, relatives and living situations. To objectify outcomes, we used the Eastern Cooperative Oncology Group (ECOG) scale as performance status measure and treatment tolerability; the Karnofsky Performance Scale to quantify the patients functional abilities; the Barthel Index to capture activities of daily living and functional independence; and the Nutritional Risk Score (NRS) to estimate the risk of malnutrition [6, 7]. Pain was assessed by Numerical Rating Scale (NRS) [8]. To allow for opioid intake comparison, an opioid calculator was used and all opioids converted to morphine equivalents either intravenous (iv) or oral (po) (www.opimeter.usz.ch).

### Team challenges

**Fig. 1:**
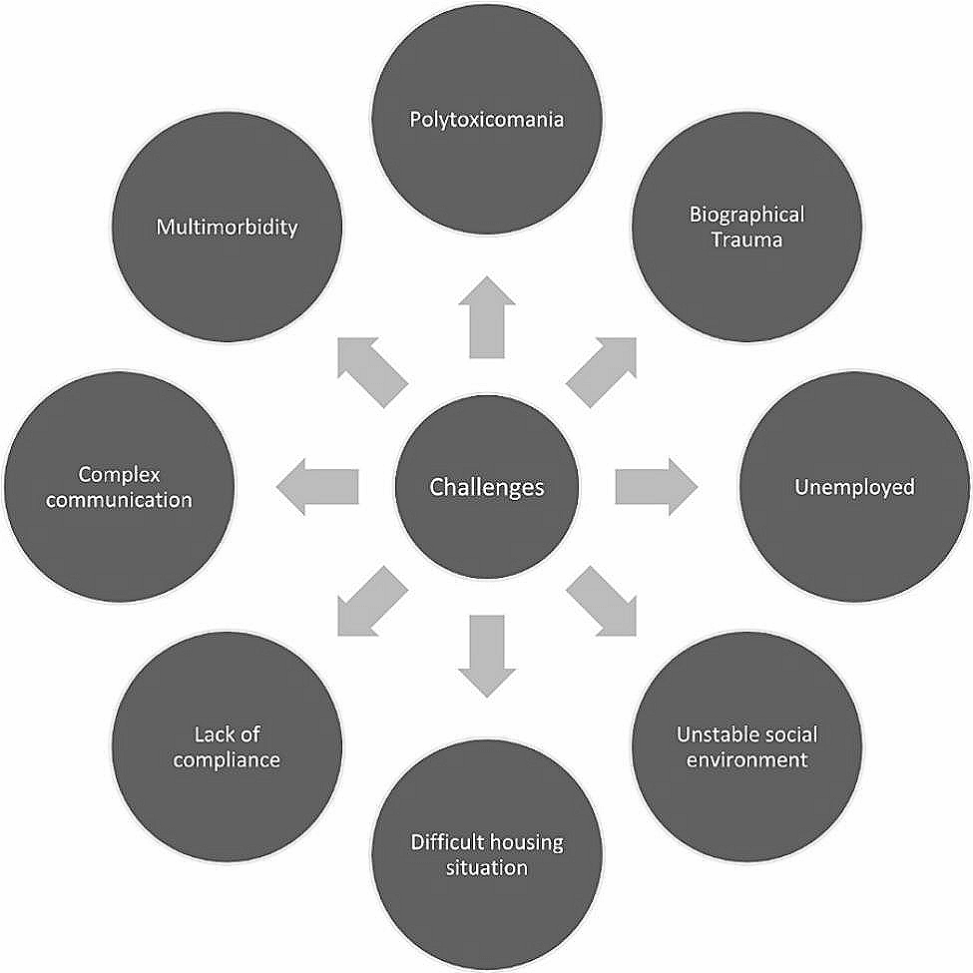
challenges

We captured all written documentations from the physicians, nursing team, social workers, psycho-oncologists and chaplains to assess specific challenges that occurred in interactions with the SUD patients. Challenges were clustered into domains after discussion within the author team comprising specialist palliative care physicians and students, a psychologist and a specialized palliative care nurse based on clinical experience and medical literature (Fig. 1). Domains were regrouped under the SENS-Model for structuring of palliative care problems (Supplementary file) (9).

### Statistics

Demographic and clinical data were analyzed by descriptive statistics on an exploratory level. We calculated mean and standard deviation for all continuous variables. The Chi-square test was performed for analysis of nominal variables between groups. For statistical analysis, SPSS Version 28 was used (SPSS IBM Corp., Armonk, NY, USA).

## Results

### Patient-related aspects

The investigated SUD patient group consisted of individuals with a median age of 52 years, range 21–79 years. The highest portion was reported to be single (56%), without children (83%), and lived either alone (39%) or in assisted living (22%). The largest portion was either unemployed or recipient of financial support (50%). (Table 1).

**Table 1:** Patient characteristics

Descriptive	Variants	N(18)	Percentage (%)
Civil status	single	10	**56%**
	married	3	17%
	divorced	2	11%
	widowed	0	0%
	unknown	3	17%
Children	yes	2	11%
	no	15	**83%**
	unknown	1	6%
Living Conditions	alone	7	**39%**
	with relatives	3	17%
	institution	2	11%
	PC institution	2	11%
	assisted living	4	**22%**
Job	unemployed	2	**11%**
	IV-recipient	7	**39%**
	office worker	3	17%
	electrician	1	6%
	construction worker	1	6%
	pensioner	2	11%
	unknown	2	11%
Place of admission	home	3	17%
	ED (emergency dep.)	6	**33%**
	institution	1	6%
	other inhouse ward	8	**44%**
Place of death	at home	1	6%
	in hospital	11	**61%**
	other institution	3	17%
	still alive	3	17%

SUD patients exhibited a wide range of substance use history. Among the substances used, nicotine was the most prevalent (88.9%), followed by opioids (66.7%) and alcohol (66.7%). Other reported drugs included cocaine, cannabinoids and sedatives.

Compared to the standard palliative care patient population, the SUD patients consisted of significantly younger patients (*p* < 0.05), and predominantly of men (*p* < 0.05) (Table 2).

**Table 2:** Group comparison

Descriptive	SUD *N* = 18 (%)	Standard *N* = 377 %	P-value
Age Median (range)	52 (21–79)	71 (21–97)	
Age Group			
≥ 70 years < 70 years	11.1% 88.9%	48.8% 51.2%	**0.002**
Gender			
Male Female	88.9% 11.1%	55.7% 44.3%	**0.005**
Cancer diagnosis			
Yes No	72.2% 27.8%	78.8% 21.2%	0.508
Discharge			
Yes No: Death on ward	38.9% 61.1%	45.9% 54.1%	0.560
Length of stay			
≥ 10 days < 10 days	60.0% 40.0%	52.5% 47.5%	0.569
Ambulatory *homecare* involved			
Yes No	38.9% 61.1%	35.6% 64.4%	0.779
Ambulatory PC service involved			
Yes No	16.7% 83.3%	27.4% 72.6%	0.316

### Functionality

Mean Karnofsky Index at admission was 48, mean ECOG performance status 2–3, both indicating a high need of support and medical assistance. Mean value of the Barthel index was 44, indicating partial assistance need in ADLs.

The average body mass index (BMI) of the examined patients lied within the normal range, as defined by the WHO, with a mean value of 20.02 kg/m2. Yet, the mean NRS was 3.6, corresponding to an intermediate level of nutritional risk, necessitating the implementation of a nutritional care plan that includes nutritional counseling.

### Co-morbidities

Aside from addiction, a considerable proportion of patients (56%) in this study experienced comorbid psychiatric conditions, encompassing anxiety disorders, depression, and schizophrenia. Furthermore, regarding physical health, liver diseases (56%), predominantly hepatitis A, B, or C (33.3%), were the most prevalent. In addition, three out of the 18 patients included in the study were diagnosed with HIV infection (17%).

### Symptoms and symptom management

**Fig. 2:**
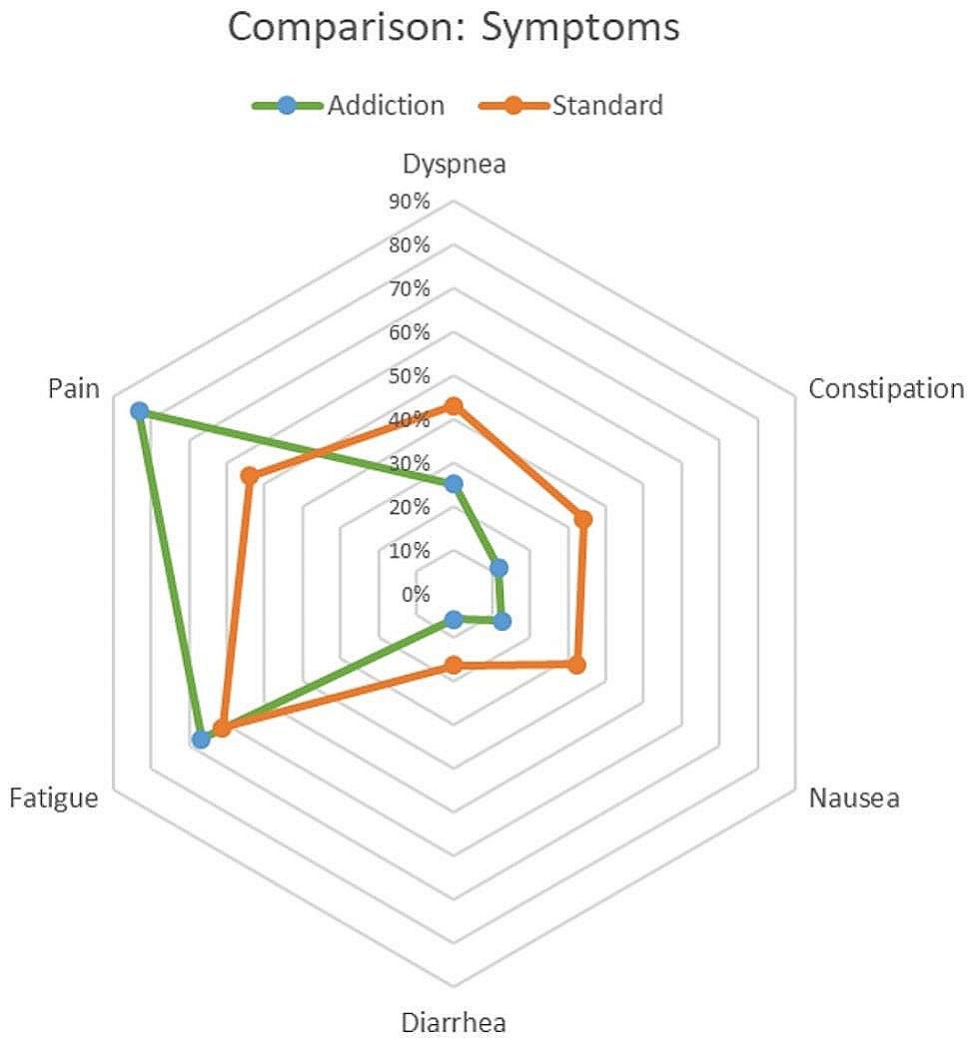
Symptom burden—comparison chart

Both SUD and standard group exhibited a high number of symptoms, including pulmonal, gastro-intestinal and pain-associated symptoms. However, SUD patients reported significantly higher pain levels (*p* < 0.05), yet significantly less nausea (*p* < 0.05). (Table 3; Fig. 2). Interestingly, both standard and SUD group experienced a decrease of pain levels during the course of hospitalization, and SUD patients never reported higher scores than 8.

**Table 3:** Symptom comparison

Symptom	SUD *N* = 18 (%)	Standard *N* = 377 %	P-value
Dyspnea			
Yes No	25.0% 75.0%	43.0%* 56.8%*	0.349
Constipation			
Yes No	11.8% 88.2%	34.2% 65.8%	0.055
Nausea			
Yes No	12.5% 87.5%	32.4% 67.6%	**0.022**
Diarrhea			
Yes No	5.9% 94.1%	16.4% 83.6%	0.245
Fatigue			
Yes No	66.7% 33.3%	61.3% 38.7%	0.674
Pain			
Yes No	83.3% 16.7%	53.9% 46.1%	**0.045**

### Substances

On average, SUD patients showed an overall usage of 1.78 (range 0–4) different types of opioids to relieve symptoms like dyspnea, pain and withdrawal symptoms, in contrast to 0.7 different types of opioids for the control cohort (range 0–3). The most used opioids were morphine (72.2%), methadone (33.3%) and oxycodone (22.2%) in the SUD cohort. The most frequently used opioids in the control cohort were morphine (30.1%), fentanyl (28.9%) and oxycodone (15.5%) as well as hydromorphone (15.1%). In the SUD cohort, the mean oral morphine equivalent per day was 637.8 mg, compared to a mean oral morphine equivalent of 114.8 mg per day in the control group (*p* < 0.001). Notably, one of the patients in the SUD cohort required on-demand doses of morphine up to 22 times per day to address the individual needs. This could however be observed in the control cohort as well, with some patients requesting up to 43 additional doses of opioids on-demand for pain control. The SUD cohort had an overall higher demand for high doses of opioids, and 61% (*n* = 11) needed daily doses of > 500 mg of oral morphin equivalent. In the control cohort, only 6% (*n* = 22) of all patients requested daily doses of > 500 mg of oral morphine equivalent per day (*p* < 0.001). Interestingly, on an individual level, the highest requested daily dose of opioids was seen in a cancer patient of the control cohort, with a daily oral morphine equivalent of 3744 mg, whereas the highest dose in the SUD group did not exceed 2062 mg of oral morphine per day.

### Challenges

SUD patients presented with complex challenges that impedes collaboration with the treating team (Fig. 1). Here, several patients reported severe trauma during life, as childhood violence, rape or experiencing death of loved ones; difficult living situations, including homelessness, several infectiological and oncological co-morbidities and complex communication with partial lack of compliance, which put a strain on the palliative care team. Three case studies demonstrate the complex patient situations exemplarily.

## Case study 1—complex communication

*BACKGROUND INFORMATION*:

*Living situation: With a colleague, who is addicted to alcohol.*

*Social situation: Her life partner died due to intoxication (Ecstasy).*

*Psychological status: Several fears: fear of dying, fear that AIDS has broken out, fear of being admitted to the psychiatric institution of University Hospital of Zurich again (was there multiple times due to decompensation and alcohol consumption); no current suicidal tendencies.*

*SUD: History of alcohol and cocaine abuse*.

*VISIT NOTE*:

- *Patient is crying, communication is not possible. The patient doesn‘t want to answer any questions.*

## Case study 2 - trauma

*BACKGROUND INFORMATION*:

*Social history: orphan; was first given to a monastery, then from foster home to foster home; knew his mother but she‘s deceased; never met his father (he was drowned in the past); knows that he has siblings but has no contact to them. His former social environment no longer exists since everyone remained in the drug scene or is already dead.*

## Case study 3—lack of compliance

*REPORT NOTE*:

- *18:30 o‘clock: patient has not been seen on ward since noon.*
- *Cannot be reached by phone and had not been seen in her home residence.*
- *Security service and police are informed.*

### Later that evening…

- *The patient has called that she has no more money left to take a cab to the hospital.*
- *The police finds her, and she seems to be under the influence of drugs but can give clear answers.*
- *3:40 o‘clock: the patient is back on ward.*
- *She‘s sitting on the edge of the bed and has very large pupils (consistent with cocaine use), anamnesis shows that she smoked crack; she sometimes talks incoherently; multiple abrasions on her neck are visible, crusted with blood.*

## Discussion

Patients suffering from addiction represent a vulnerable, yet under-researched group of patients in palliative care, with high symptom burden and several needs.

Here, we report on a group of patients with severe SUD treated in our palliative care department. We confirm that the SUD cohort comprises significantly younger patients compared to the standard group, pointing towards an overall lower life expectancy and higher incidence of life-threatening diseases at earlier ages that individuals with SUD have to cope with. While these patients were younger, they were still more socially isolated compared to standard populations, with the majority being single, living alone, and having no children (Table 1). This underscores the frequent lack of stable network and support systems for SUD patients. The small number alone of patients identified here underlines the social marginalization and underrepresentation and by that, the vulnerability of a patient group that presents with severe morbidities and unmet needs. Multiple studies in the current literature have consistently demonstrated that individuals with SUD possess distinct requirements concerning social and psychological support. Establishing an appropriate social environment can serve as a protective factor. However, patients with SUD are more prone experiencing familial conflicts, financial strain, and relationship breakdowns compared to the general population. Our patients also reported traumata, like childhood violence, or sexual harassment, in their history. Moreover, these patients are prone to be unemployed or of lower socioeconomic groups [5], also shown in our cohort, where it is worth noting that 38.9% of our SUD patients were recipients of disability pension (Table 1), a significantly higher percentage compared to the general population of Switzerland, which stood at 4% in December 2020 according to the Swiss Federal Statistical Office.

With regard to comorbidities, our SUD group contained a noteworthy number of patients who had HIV, liver diseases (specifically hepatitis A, B, or C), and psychiatric comorbidities. These findings align with existing literature, which indicates that intravenous drug use is a prevalent risk factor for HIV exposure [10]. Consecutively, the presence of immunodeficiency renders patients more vulnerable to additional diseases, leading to a higher burden of comorbidities on top of SUD. Consequently, many opioid addicts experience accelerated biological aging [11]. However, beyond somatic comorbidities, psychiatric diagnoses beyond SUD, such as depression, anxiety, and schizophrenia, are often seen in SUD patients as dual diagnoses, and suicide risks are higher in persons with SUD [5, 12, 13]. In our SUD population, more than half of the patients suffered from psychiatric conditions, mainly anxiety, depression and schizophrenia, confirming an additional need for comprehensive care and specific skills.

Within the currently existing literature, three main symptoms have been commonly observed in palliative care patients: pain, dyspnea, and anxiety/agitation [11]. Among these, pain and dyspnea were the most frequently reported symptoms in both our SUD and standard group, with pain being reported at significantly higher levels in the SUD group (Table 3). This is in line with previous reports that patients with alcohol dependence report higher level of pain [5]. This phenomenon can also be seen in patients with a history of injection drug use. Moreover, chronic opioid users frequently experience opioid tolerance and opioid-induced hyperalgesia, contributing to increased pain sensitivity [10, 14]. Additionally, pain may also emerge in the context of withdrawal symptoms [5, 11]. Our findings regarding pain development during hospitalization align with these observations, as a higher percentage of patients in the SUD group, compared to the standard group, reported Numeric Rating Scale (NRS) scores exceeding 5 upon admission and during last contact. This is of utmost importance especially for SUD patients, because less trained clinicians may under-prescribe analgesics to individuals with SUD or non-opioid analgesics that are less prone to abuse but unfortunately also less effective [10], eventually leading to undertreatment of this vulnerable group [15, 16]. With regard to the control cohort of standard palliative patients, while the mean oral morphine equivalent was higher in SUD patients with a mean oral intake of 637.8 mg per day, the highest opioid dose was needed by an elderly female patient with no history of substance abuse in severe pain; and several patients of the control cohort on patient-controlled analgesia (PCA) pumps requested up to double the number of on-demand boli in the standard patient group compared to SUD patients. This strongly indicates that pain is the leading signal to provide analgesia in any person, and according to the primary goal of palliation—to alleviate burden in a patient-centered manner—this should be independent of the primary disease or diagnosis. The treatment of chronic pain with opioids contributed to the development of an opioid crisis and severe addiction problems in several countries and has rightly raised concerns and fears about opioid prescriptions in Switzerland as well [17]. However, the right to pain relief that originated in palliative and end-of-life care should not be overruled in this specific context with a different time frame and clinical context [18]. In palliative and end-of-life care, unlike chronic pain, tapering of opioids is not the primary goal of care. Potential side effects of higher opioid doses, as fatigue, are balanced against pain as a symptom, ideally in a joint decision with the patient, and advance directives usually include these considerations.

The only other symptom that reached significance in group comparison was nausea (*p* = 0.022) (Table 3), with patients without SUD displaying a higher prevalence. Various factors may contribute to this observation; however, one may postulate that SUD persons develop a tolerance and adaptation to this symptom.

With regard to functional status, our SUD cohort displayed an average Barthel Index score of 43.8 and an average KPS score of 48.1, indicating that more than half of the investigated patients required partial assistance from healthcare professionals for their activities of daily living. Comparing these results to a study from 2022 examining 220 cancer patients in a palliative care setting with a mean Barthel Index score of 72.6 and KPS score of 61.7, we reveal that the patients in our SUD group required greater assistance than standard palliative care patients [19]. Interestingly, while our SUD patient cohort presented with a better functional ECOG score at admission compared to our standard group, they were still more likely to die on ward, and to die younger compared to the standard group. However, finding a suitable discharge location for those who left the hospital was challenging due to the fact that nursing homes or hospices were often neither prepared to deal with such high amounts of opioids, nor with the psychiatric co-morbidities; whereas the assisted living homes for SUD persons could not cope with the often oncological, progressing disease.

Finally, with regard to challenges in this specific patient group, we demonstrate that our SUD patients, as described in the literature, exhibit maladaptive coping strategies [5]. Moreover, patients actively engaged in substance use often present challenges in their willingness to participate in treatment and display reduced compliance [20]. Numerous examples highlight this problem, such as missed appointments, difficulties in contacting patients, denial to communicate with healthcare providers, institutional distrust, and an overload of decision-making [11]. These factors collectively contribute to the complexity of interacting with these patients. Partly, self-protection may also be a contributing factor, particularly when individuals have experienced disappointments within their social surroundings. As a result, they constructed emotional barriers, making them less responsive to external assistance [21]. In the case of SUD, motivational interviewing and leaving room for the patients’ ambivalent opinions are usually tools of communication; however, motivational interviewing, as an established communication tool in addiction treatment, encompasses other goals as those classically used in the setting of palliative care (e.g. shared decision making), which makes communication challenging for both the team and the patient.

Our study has limitations. First, the data collection was limited by the retrospective nature of physician and health care worker reports, and we could not contribute the patient view here. Second, the SUD group only includes 18 people, which represents a rather small number, albeit being to date a quite unique cohort in palliative care in Switzerland. This underlines the underrepresentation and unmet needs of this vulnerable population especially in the late palliative phase of life. In addition, the comparison cohort consists of more than 300 people, making it even challenging to compare those two groups. However, a matched cohort would have precluded the identification of characteristic factors, as age and gender. Another limitation of this analysis is the descriptive nature of the data, comprising exploratory analyses, and the consideration of multiple testings, that increases the likelihood of overestimated findings, which is why the results should be interpreted with caution. Finally, the challenges described were based on mainly subjective experiences that were reported from health care practitioners and physicians. Lastly, as there is little information in the current literature about patients with SUD in a palliative setting, it was not possible to find comparative information about the situation of other SUD patients in palliative care in Switzerland. Therefore, the results of this work had to be discussed in the context of current research from other countries, as the USA, which might induce biases due to different health care systems. This shows once again why it is important to continue research in this field.

We feel it is crucial to address this topic to gain knowledge and routine in working with SUD patients in palliative care, as this group will probably increase over the years due to the commendable harm-reduction policy of Switzerland and the substantial rate of SUD patients undergoing opioid agonist therapy [22]. Indeed, opioid agonist therapies may be associated with reduced, potentially dangerous opioid co-use [23]. However, even in a wealthy country, there is still a care gap for this patient group, with regard to treatment, but also care institutions in the last phase of life, due to the complex and multiple symptoms. Given the understanding that individuals with SUD are particularly prone to a range of psychological and physical diseases, it becomes crucial to enhance the interprofessional teamwork within palliative care for vulnerable groups, and to strengthen the network between psychiatrists and palliative care specialists.

### Electronic supplementary material

Below is the link to the electronic supplementary material.

Supplementary Material 1

## Data Availability

The dataset supporting the conclusions of this article is available from the corresponding author upon reasonable request.

## Code Availability

Not applicable for this publication.
