# Supplementary material for: Characteristics and clinical challenges in patients with substance use disorder in palliative care—experience from a tertiary center in a high-income country
Source: BMC Palliat Care. 2024 Jan 30;23:28. doi: 10.1186/s12904-024-01366-x (PMC10826251; doi:10.1186/s12904-024-01366-x)
Supplement: Supplementary file 1 — Supplementary Material 1 [file 12904_2024_1366_MOESM1_ESM.pdf]

Supplementary file: Data assessment script

Main question: what are the most important issues occurring with substance use disorders in palliative care?

Patient data assessment script

1) ***Baseline variables:***

- Age
- Gender
- Substance(s) used
- Main disease
- Co-morbidities
- Main symptoms reported
- Numerical rating scale
- Eastern Cooperative Oncology Group (ECOG) Performance status
- Karnofsky-Index
- Barthel Index
- Nutritional Risk Score

2) ***Additional and specific Challenges (based on palliative care SENS – Modell*)***

- Review of written information on specific challenges while providing palliative care in the SUD cohort.
- Discussion of relevant and most frequent items identified as challenges for either patient; team; or both.
- Clustering of recurrent problems (> 3 occurrences) into 10 main domains
- Regrouping of main domains under the palliative care SENS structure
- Review all SUD patient charts to re-assess occurrence of identified domains

Symptoms
- Personality (disorders)
- Multimorbidity
- Polytoxicomania

*"**E**ntscheidungsfindung"* / Decision-making
- (complex) communication
- (lack of) compliance
- Bibliographical trauma

Network
- (unstable) social environment
- Lack of family

Support
- (Un)employment
- (difficult) housing situation

* Eychmüller, S. SENS is making sense - on the way to an innovative approach to structure Palliative Care problems. Ther Umsch. 2012; 69:87-90.
